# Cerebellar Asymmetry of Motivational Direction: Anger-Dependent Effects of Cerebellar Transcranial Direct Current Stimulation on Aggression in Healthy Volunteers

**DOI:** 10.1007/s12311-023-01644-z

**Published:** 2024-01-04

**Authors:** Eline S. Kruithof, Jana Klaus, Dennis J. L. G. Schutter

**Affiliations:** Department of Experimental Psychology, Helmholtz Institute, Utrecht University, Heidelberglaan 1, 3584 CS Utrecht, The Netherlands

**Keywords:** Aggression, Anger, Cerebellum, Heart rate, State-dependency, Transcranial direct current stimulation

## Abstract

It has recently been theorized that the frontal asymmetry of approach- and avoidance-related motivation is mirrored in the posterolateral cerebellum. Accordingly, left-to-right dominant cerebellar activity is associated with avoidance-related motivation, whereas right-to-left dominant cerebellar activity is associated with approach-related motivation. The aim of this study was to examine the cerebellar asymmetry of motivational direction in approach-related behavior in the context of aggression. In this randomized double-blind sham-controlled crossover study, thirty healthy right-handed adult volunteers received 2 mA active or sham left cathodal-right anodal transcranial direct current stimulation (tDCS) to the cerebellum on two separate occasions while engaging in the Point Subtraction Aggression Paradigm (PSAP) task to measure aggressive behavior. Self-reported state anger was assessed before, halfway and immediately after the task, and heart rate and heart rate variability (HRV) were measured during the task. No main effects of tDCS on aggressive behavior, heart rate and HRV were found. Higher state anger before and during the PSAP task was associated with increased aggressive behavior in the active compared to sham tDCS condition. Aggressive behavior was positively correlated with heart rate during active tDCS, while an inverse association was observed during sham tDCS. Results provide support for the cerebellar asymmetry of motivational direction in approach-related behavior and illustrate the importance of affective state-dependency in tDCS-related effects.

**Supplementary Information:**

The online version contains supplementary material available at 10.1007/s12311-023-01644-z.

## Introduction

According to the motivational direction model of frontal asymmetry, left-to-right dominant frontal cortical activity is associated with approach-related motivation, whereas right-to-left dominant frontal cortical activity is associated with avoidance-related motivation [1–3]. Approach-related motivation refers to the tendency to go towards a stimulus and is motivated by obtaining a reward [4, 5]. By contrast, avoidance-related motivation refers to the tendency to move away from a stimulus and is motivated by avoiding punishment [4].

In agreement with the motivational direction model, the approach-related emotion anger as well as aggressive behavior have been linked to a left-lateralized (i.e., left-to-right dominant) frontal asymmetry [6–9]. Aggression can be defined as behavior directed towards harming or injuring another individual who is motivated to avoid threat [10]. Anger is an emotion which can be elicited in response to perceived threat, provocation or frustration [11, 12] and increases the likelihood of aggression [13]. In a previous study, transcranial direct current stimulation (tDCS) was administered in healthy volunteers to examine the relationship between frontal asymmetry, anger and aggression [8]. First, participants were asked to write an essay on a controversial topic. Participants were then randomly allocated to receive 15 minutes of either left anodal-right cathodal tDCS, left cathodal-right anodal tDCS or sham tDCS to the frontal cortex. After tDCS, participants received insulting feedback on their essay from another (fictional) person and were able to aggress to the insulting person during a competitive task by administering noise blasts. Results showed that higher levels of state anger evoked by the insulting feedback were associated with administering more noise blasts in the left anodal-right cathodal tDCS condition exclusively. This study showed that state anger in combination with approach-related motivational tendencies, arguably by increasing left and decreasing right frontal cortical activity with tDCS [14], can facilitate aggressive behavior in healthy volunteers.

A brain structure that is increasingly being acknowledged for its involvement in aggression is the cerebellum [15, 16]. Electrical stimulation of the deep cerebellar nuclei (DCN) in cats [17, 18] as well as indirect stimulation of the DCN by optogenetic inhibition of Purkinje cell activity in mice [19] increased attack behaviors and aggression-related autonomic activity. Furthermore, human lesion studies have linked cerebellar damage, particularly to the vermis, to emotion regulation deficits including aggression, irritability and impulsivity [20, 21]. More recently, a functional neuroimaging meta-analysis showed that anger and threat processing involve right Crus I/II and aggression involves bilateral lobules V and VI [12]. Furthermore, a recent fMRI study in healthy volunteers showed that aggressive behavior could be localized to the right cerebellar posterolateral lobe [22].

In line with the contralateral cerebello-cortical connections [23, 24], functional asymmetries underlying non-motor functions in the cerebral cortex are also present in the cerebellum, but in a reversed manner [25]. As such, it can be theorized that a cerebellar functional asymmetry as reflected by left-to-right dominant activity is associated with avoidance-related motivation, whereas right-to-left dominant activity is associated with approach-related motivation [26].

Both anger and aggression are associated with short-term changes in cardiac activity. Anger is related to increases in heart rate and reductions in heart rate variability (HRV; [27]). Aggression is associated with increased heart rate relative to baseline levels in response to negatively valenced stimuli [28]. Furthermore, elevated heart rate was observed in a group of female adolescents that were provoked more by and behaved more aggressively towards an opponent in a competitive task than the control group [29]. In addition, aggression towards a provoking opponent has been associated with a lower HRV [30]. Increased heart rate and reduced HRV can result from activation of the sympathetic branch and/or diminished activation of the parasympathetic branch of the autonomic nervous system [31, 32]. Relatively dominant sympathetic nervous system activity is part of the fight-flight response that allows an organism to act in the face threat [31].

The aim of the present double-blind sham-controlled crossover study was to examine the cerebellar asymmetry of motivational direction in approach-related behavior in the context of aggression. To this end, left cathodal-right anodal tDCS was applied to the posterior cerebellum of healthy volunteers. We hypothesized that tDCS-induced right-to-left dominant cerebellar activity would facilitate aggressive behavior during a provocation task. Furthermore, analogous to the study of Hortensius et al. [8], we expected that the effect of tDCS on aggressive behavior would be positively modulated by the participant’s state anger. Additionally, we explored possible relationships between cerebellar tDCS and autonomic activity as measured with heart rate and HRV.

## Materials and Methods

### Participants

Thirty healthy, right-handed, non-smoking adult volunteers (24 females, mean age = 23.02 years, *SD* = 3.18, range = 19–32) participated in the study in exchange for course credit or a monetary compensation of 16 EUR. They had no history of neurological or psychiatric conditions, family history of epilepsy, skin disease or allergy, heart disease, metal in the head, pacemaker or neurostimulator, were not pregnant and did not use medication (except for oral contraceptives). The required sample size was estimated with G*Power 3.1.9.4 [33] for a repeated measures ANOVA with the following settings: *f* = 0.275 (based on [34]), α = 0.05, power = 0.80, number of groups = 2, number of measurements = 2, correlation among repeated measures = 0.5, and yielded a sample size of 30 participants. All participants gave written informed consent. The study was approved by the ethics committee of the Faculty of Social and Behavioural Sciences of Utrecht University.

### Provocation Task

A modified version of the Point Subtraction Aggression Paradigm (PSAP; originally designed by Cherek, [35]) programmed in E-prime version 2.0 (Psychology Software Tools) was used to measure aggression in response to provocations by a fictional opponent. Participants were led to believe that they would play against an actual opponent. The aim of the computer game was to gain as many points as possible and participants were told that all points would be exchangeable for money (i.e., three cents for every point collected). A point could be earned by pressing the 1 key 50 times (i.e., earn option). Participants were informed that they may see their point counter flash several times in red font throughout the task, implying that the opponent had stolen a point from them (i.e., they were provoked). By pressing the 2 key ten times, participants could steal a point from their opponent (i.e., steal option). By pressing the 3 key ten times, participants protected their points from being stolen for 30 s (i.e., protect option). Participants were told that they would be randomly assigned to the condition in which their opponent could keep the stolen points, whereas they could not keep the points stolen from their opponent. Because stealing does not benefit the participant financially, it can be regarded as behavior intended to harm the opponent [36]. While conventional versions of the PSAP require participants to complete a fixed number of key presses associated with a particular action (i.e., earning, stealing or protecting) before being able to select another option (e.g., [36–40]), our version allowed participants to switch between options before completing this number of key presses. When switching to another option before completion, participants could return to the earlier chosen option whenever they wanted and continue from the number of keys pressed earlier. This opportunity allowed participants to immediately respond to provocations by the opponent. The first provocation was programmed to occur 30 s after the start of the task. Completing ten key presses of either the steal or the protect option initiated a provocation-free interval of 30 s. Participants were informed about the protective effect (i.e., the initiation of a provocation-free interval) of the protect but not the steal option. After the first provocation, subsequent provocations occurred randomly within 6 and 45 s unless a provocation-free interval was initiated. Provocations were preprogrammed to occur at least once per minute. Participants completed a practice round of 1 min before engaging in two 5-min blocks of the task.

### Transcranial Direct Current Stimulation

Bipolar electric stimulation was applied via two rubber electrodes (5 × 5 cm) placed in sponges covered with conductive gel using a battery-driven DC stimulator (NeuroConn GmbH, Ilmenau, Germany). The electrode sponges were placed under an EEG cap for appropriate localization and fixation. The cathode was positioned over the left cerebellum corresponding to electrode position P9 in the international 10–10 EEG system and the anode was positioned over the right cerebellum corresponding to electrode position P10 in the international 10–10 EEG system. Active cerebellar tDCS was delivered for 15 min at a current intensity of 2 mA (current density: 0.08 mA/cm^2^, total charge density: 72 mC/cm^2^) with a 30 s ramp-up and ramp-down phase at the start and end of the stimulation period, respectively. In the sham cerebellar tDCS condition, participants received active stimulation for 30 s after an initial 5-s ramp-up, followed by a ramp-down of 5 s. Cerebellar tDCS was delivered while participants filled out the questionnaires and engaged in the task. The impedance of the electrodes was kept below 10 kΩ throughout the stimulation.

Order of stimulation condition was counterbalanced and randomized across participants. The experimenter entered a pre-assigned four-digit code into the DC stimulator that unbeknownst to the experimenter and the participant would initiate active or sham cerebellar tDCS.

### State Anger

The state anger scale of the State-Trait Anger Expression Inventory-2 (STAXI-2) was used to assess state anger [41]. The scale consists of ten items to which participants responded on a ten-point scale ranging from ‘not at all’ to ‘very much so’. Items appeared on the screen one by one and were presented in random order. A higher score on the state anger scale of the STAXI-2 corresponds to a higher level of state anger.

### Electrocardiography

Electrocardiography was performed using a BioSemi ActiveTwo system (BioSemi, Amsterdam, The Netherlands) at a sampling rate of 2048 Hz. Electric signals from the heart were detected by three Ag/AgCl electrodes filled with conductive gel. The first active electrode was placed under the right collarbone, the second active electrode was placed under the lowest left rib and a ground electrode was placed on the right abdomen.

### Procedure

Participants were told that the aim of the study was to investigate the role of the cerebellum in gameplaying. They were invited to the lab two times for approximately 45 min each. The two sessions occurred at the same time of the day and there were at least seven days in between. In the days before the first session, participants received the study information letter and were asked to fill out the screening form to check for contraindications of tDCS. Participants were requested not to drink coffee or tea or to eat chocolate in the two hours before the test session and not to consume alcohol 24 h in advance. At the start of the test session, participants had the opportunity to ask questions and then signed the consent form. To contribute to the credibility of playing against an actual opponent, the lab in which the study was conducted consisted of two separate, but adjacent rooms. The participant was situated in one room while the door of the adjacent room was kept closed to create the impression that the opponent was already there. First, the electrodes for the electrocardiography were applied. Next, the EEG cap was placed over the participant’s head and centered at the vertex, after which the tDCS electrodes were applied and stimulation was started. The state anger scale of the STAXI-2 was administered before, during, and immediately after the task. To gauge the effectiveness of the deception, participants answered two open questions about their ideas on the opponent’s age and gender after the second session. One out of 30 participants indicated on one of the questions that the opponent might have been a computer. At the end of the second session, participants were debriefed about the purpose of the study and, in order to check whether blinding was successful, were asked to guess in which session they had received active tDCS. After completion of the second session, all participants received the same amount of money (i.e., 2.50 EUR per session) in addition to their participation fee, independent of the number of points earned during the task.

### Data Reduction and Statistical Analysis

For the behavioral analyses, aggression was scored as the number of key presses on the steal option corrected for the total number of key presses and the number of provocations, scaled by 1000 (i.e., [1000 × No. key 2 presses]/[No. of total key presses × No. of provocations]; [40]). Overall state anger scores before, during and after the task were calculated by dividing the sum of scores by ten (the number of items) and were averaged across the sham and active tDCS condition due to non-significant differences between conditions (*ps* ≥ 0.073).

To test whether the task was successful at inducing anger, a repeated measures ANOVA was performed to assess self-reported anger pre-, during- and post-task in the sham tDCS condition. A Greenhouse-Geisser correction for non-sphericity was applied. To investigate whether aggression throughout the task differed between the active tDCS and sham tDCS condition, an additional repeated measures ANOVA was performed with tDCS condition as within-subjects factor. To examine whether the effects of tDCS condition were different depending on state anger levels before, during and after the task, these variables were added as covariates in separate repeated measures ANCOVAs. Additionally, the analyses involving the effect of cerebellar tDCS on aggression were performed after outlier exclusion. For the main effect of tDCS condition on aggression, outliers were identified based on scores ± 3 *SD* from the mean for each tDCS condition. For the interactions between tDCS condition and pre-, during- and post-task state anger on aggression, outliers were identified based on standardized residuals in the linear regression for each tDCS condition that were larger than 3 [42].

For the heart rate and HRV analysis, BrainVision Analyzer version 2.1 (Brain Products GmbH) was used to identify the R peaks after applying a 30-Hz high-pass filter. Each session was segmented into 15 s segments. For each segment, an average heart rate (in beats per minute) and the interbeat intervals (IBIs; in ms) were calculated. The root mean square of successive differences (RMSSD) parameter of HRV was calculated in MATLAB R2020b (MathWorks) using the IBIs. Three participants were excluded from the heart rate analysis due to procedural or technical issues. Outliers in the heart rate and HRV data (± 3 *SD* from the individual mean) were removed from the analysis (heart rate data: 0.001%; HRV data: 0.016%). Individual aggression scores were calculated for each 5-min block of the task according to the same formula used for the behavioral analysis. Additionally, state anger change scores were computed for every 5-min block of the task, reflecting the change in state anger from pre- to during-task (i.e., after the first block; during- minus pre-task) and from during- to post-task (i.e., after the second block; post- minus during-task). Instead of using absolute pre- and during-task state anger scores, state anger change scores were used in this analysis to reflect task-induced modulations in state anger.

Heart rate and HRV during the task were analyzed with linear mixed models using the lme4 package version 1.1–27.1 [43]. The analysis included the fixed effects tDCS condition, state anger change, aggression, and their interactions as well as by-participant intercepts and by-participant slopes for the effect of tDCS condition. Linear mixed models were checked for the presence of outliers using Cook’s distance and were not identified. Heart rate was both log-transformed and squared and HRV was log-transformed only to achieve homoscedasticity.

All statistical analyses were performed in R version 4.1.2 (R Core Team, 2021). The alpha level of significance was set to 0.05 (two-tailed) for all analyses.

## Results

Cerebellar tDCS was well tolerated. Blinding score data was available for a subset of participants (*N* = 23) and indicated that blinding was effective (*Χ*^2^_1_ (23) = 0.048, *p* = 0.827). A repeated measures ANOVA showed that state anger increased significantly during the task in the sham tDCS condition (*F*(1.28, 37.22) = 4.535, *p* = 0.031), indicating that the task successfully elicited anger. Table 1 shows the descriptive statistics of the task and state anger scores per tDCS condition for the behavioral analysis. Table 2 summarizes the descriptive statistics of the cardiac measures and state anger change scores per tDCS condition for the heart rate and HRV analysis.

**Table 1:** Descriptive statistics of the task and state anger scores per tDCS condition for the behavioral analysis (*N* = 30)

	Sham tDCS (*M* ± *SD*)	Active tDCS (*M* ± *SD*)
Aggression	4.05 ± 3.21	4.22 ± 3.51
Number of provocations	15.5 ± 2.53	15 ± 2.51
Pre-task state anger	0.31 ± 0.49	0.53 ± 0.71
During-task state anger	0.74 ± 1.14	0.82 ± 0.95
Post-task state anger	0.61 ± 0.61	1.09 ± 1.58

**Table 2:** Descriptive statistics of the cardiac measures and state anger change scores per tDCS condition for the heart rate (in beats per minute) and HRV (in milliseconds, according to the root mean square of successive differences) analysis (*N* = 27)

	Sham tDCS (*M* ± *SD*)	Active tDCS (*M* ± *SD*)
Heart rate	83.30 ± 12.25	83.40 ± 13.58
HRV	37.95 ± 27.27	37.44 ± 28.08
State anger change (during-pre task)	0.50 ± 0.98	0.29 ± 0.80
State anger change (post-during task)	0.07 ± 0.60	0.08 ± 0.52

HRV = Heart rate variability

**Fig. 1:**
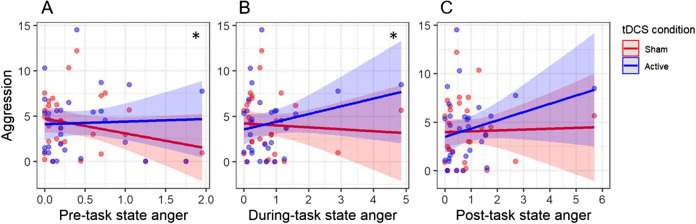
The interactions between tDCS condition and pre-, during- and post-task state anger levels on aggression. Asterisks indicate a significant interaction (*p* < 0.05)

The repeated measures ANOVA revealed no main effect of tDCS condition on aggression (*F*(1,29) = 0.158, *p* = 0.694). One outlier was identified in the active tDCS condition for the participant that had the highest aggression score in the study sample. Removing this participant from the analysis did not change results for the main effect of tDCS condition on aggression (*F*(1,28) = 0.051, *p* = 0.823). The repeated measures ANCOVAs showed significant interactions between tDCS condition and pre-task (*F*(1,28) = 5.652, *p* = 0.025; Fig. 1A) as well as during-task state anger levels (*F*(1,28) = 6.253, *p* = 0.019; Fig. 1B) on aggression. Higher self-reported state anger before and during the task was related to increased aggressive behavior in the active compared to sham cerebellar tDCS condition. Follow-up analyses indicated no association between pre-task state anger and aggression during active (*r*(28) = 0.043, *p* = 0.822) and sham tDCS (*r*(28) = -0.266, *p* = 0.156). Additionally, no association was found between during-task state anger and aggression during active (*r*(28) = 0.238, *p* = 0.206) and sham tDCS (*r*(28) = -0.065, *p* = 0.732). The interaction between tDCS condition and post-task state anger levels on aggression was marginally significant (*F*(1,28) = 3.864, *p* = 0.059; Fig. 1C). Follow-up analyses indicated no association between post-task state anger and aggression during active (*r*(28) = 0.270, *p* = 0.149) and sham tDCS (*r*(28) = 0.031, *p* = 0.872). Two outliers were identified for the association between during- and post-task state anger and aggression in the active tDCS condition that concerned the same participant. This participant had the highest aggression score in the study sample in combination with a low state anger score. Removing this participant from the analyses did not change results for the interaction between tDCS condition and pre- (*F*(1,27) = 5.681, *p* = 0.024), during- (*F*(1,27) = 6.529, *p* = 0.017) and post-task state anger levels (*F*(1,27) = 4.162, *p* = 0.051) on aggression.

**Fig. 2:**
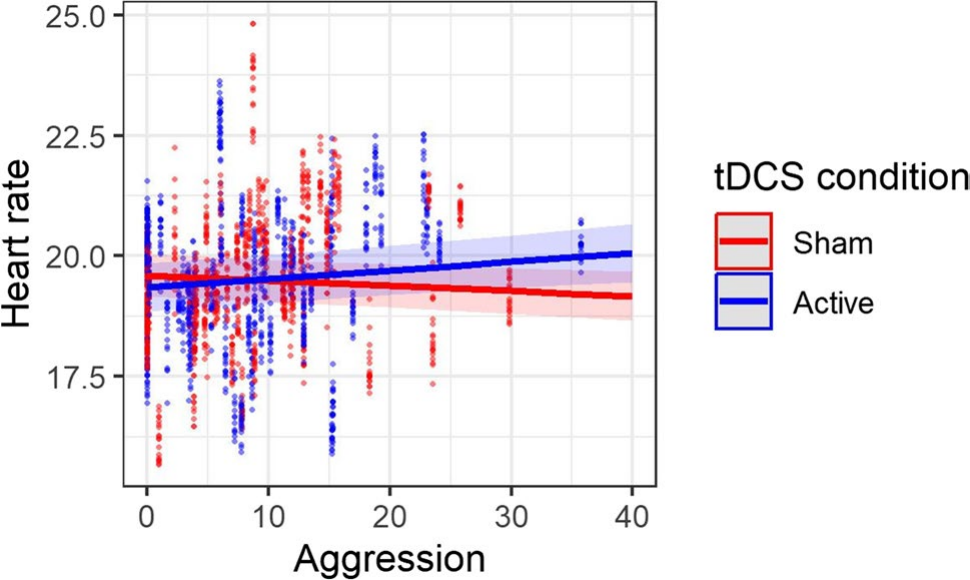
Heart rate (log-transformed and squared) as a function of aggression and tDCS condition

For heart rate, the linear mixed effects model revealed a positive main effect of state anger change (*β* = 0.094, *SE* = 0.024, *t* = 3.99, *p* < 0.001) and an interaction between tDCS condition and aggression (*β* = -0.014, *SE* = 0.004, *t* = -3.48, *p* = 0.001; Fig. 2). Aggression was positively related to heart rate during active tDCS, compared to an inverse association during sham tDCS.

HRV decreased as a function of state anger change (*β* = -0.078, *SE* = 0.019, *t* = -4.07, *p* < 0.001). A complete documentation of the linear mixed effects models results for heart rate and HRV can be found in the supplementary materials.

## Discussion

Results showed no main effect of cerebellar tDCS on aggressive behavior. However, higher state anger levels before and during the task were associated with increased aggressive behavior during active as compared to sham cerebellar tDCS. Active tDCS thus facilitated the transition from anger to aggression, supporting the idea that right-to-left dominant cerebellar activity is associated with approach-related motivation. The notion of relative right cerebellar involvement in approach-related motivation concurs with results from recent structural [16] and functional [22] MRI studies and a functional neuroimaging meta-analysis [12] that reported right posterior cerebellar involvement in aggression. Importantly, it should be noted that the interaction between tDCS condition and baseline state anger was driven by a state anger-dependent decline in aggressive behavior during sham tDCS, while this association levelled off during active tDCS. By contrast, the interaction between tDCS condition and state anger during the task was driven by a state anger-dependent increase in aggression in the active tDCS condition, while there was no association between state anger and aggressive behavior during sham tDCS. Lower aggression with higher levels of state anger before and during the task in the sham tDCS condition may reflect a strategic anger response where the monetary reward is preferred over the option of responding aggressively. Participants in the active tDCS condition, however, seem to dismiss such a strategy when taking into account state anger during the task (i.e., the emotional response induced by the provocation task). This may suggest a shift in the cost-benefit analysis from focusing on monetary rewards during sham tDCS towards behaving aggressively during active tDCS, as a result of the tDCS-induced approach motivation. With regard to state anger levels after the task, participants in the active tDCS condition still showed higher aggression with higher levels of state anger in comparison to lower aggression in the sham tDCS condition. However, this interaction was only marginally significant, indicating a fading out of the anger effect after the task had ended. For the frontal cortex, Hortensius et al. [8] showed that a higher level of self-reported anger was associated with an increase in aggression following left anodal-right cathodal tDCS. In line with this finding, we observed a state anger-dependent effect of cerebellar tDCS on aggression when manipulating cerebellar activity using a reversed bipolar tDCS montage. Importantly, it should be mentioned that the significant results of a pre- and during-task state anger-dependent increase in aggression in the active compared to sham tDCS condition were not corrected for multiple testing and therefore, these results should be treated with caution. However, our findings concur with the previous finding of Hortensius et al. [8], and add to the increasing body of evidence that points towards the relevance of affective (psychological) states in the effects of tDCS on behavior [44].

Aggression was positively related to heart rate during active tDCS, while an inverse association was observed during sham tDCS. Empirical studies have established a role of the cerebellum in modulating cardiac activity [17, 45–55]. Importantly, we found no main effect of tDCS but an aggression-dependent tDCS effect on heart rate. The posterolateral cerebellum as targeted in this study is considered part of the salience network [56] and has been implicated in processing interoceptive and autonomic signals [57], which can affect emotional states [58, 59]. Active tDCS may have facilitated access of interoceptive heart rate signals to the cerebellum and contributed to aggressive behavior. According to this idea, the role of the cerebellum is interpreted from an embodied perspective in the context of emotional and motivational behavior [60, 61]. The present study does not allow to make any inferences about the underlying process by which the cerebellum influences behavior based on interoceptive heart rate signals and anger states. This question remains to be elucidated in future research.

No evidence was found that cerebellar tDCS influenced HRV or that HRV modulated the effects of cerebellar tDCS on aggressive behavior. The RMSSD parameter of HRV as used in this study is a measure of vagal tone [62], while heart rate is affected by both sympathetic and parasympathetic (vagal) input [31]. Arguably, the modulation of aggressive behavior in the active tDCS condition based on cardiac signals might be mainly sympathetically driven, thus rendering a tDCS-induced change in HRV unlikely.

It should be noted that the results might not be representative for males as 80% of the study sample consisted of female participants. For the cerebrum, several studies have demonstrated sex differences in the effects of tDCS on cortical excitability, electric field modeling and task-related functions [63–68]. For example, these differences have been suggested to involve sex-dependent variability in hormones, brain anatomy and neurophysiology [63, 68–72]. However, to the best our knowledge, how sex differences contribute to cerebellar tDCS effects remains unknown, also in the context of aggression. Future research is needed to clarify this.

The observation of a state anger-dependent effect on aggression during left cathodal-right anodal cerebellar tDCS can be interpreted as a cerebellar asymmetry in terms of an increase in approach- relative to avoidance-related motivation. However, our findings can also be explained by a decrease in avoidance- relative to approach-related motivation. According to the motivational direction theory of cerebellar asymmetry, left-to-right dominant cerebellar activity is associated with avoidance-related motivation. As such, cerebellar left anodal-right cathodal tDCS is hypothesized to facilitate avoidance-related behavior, arguably causing participants to ignore provocations and invest more in earning points instead of behaving aggressively [73]. Future research is needed to test this hypothesis. Additionally, further research should clarify the distinct contributions of the left and right cerebellum to motivational tendencies and aggression.

## Conclusion

This study provides support for the cerebellar asymmetry of motivational direction in approach-related behavior and illustrates the importance of affective state-dependency in tDCS-related effects.

### Supplementary Information

Below is the link to the electronic supplementary material.

Supplementary file1 (DOCX 16 KB)

## Data Availability

The data used for this article is available at https://osf.io/pqwhn/.
