# Supplementary material for: Cerebellar Asymmetry of Motivational Direction: Anger-Dependent Effects of Cerebellar Transcranial Direct Current Stimulation on Aggression in Healthy Volunteers
Source: Cerebellum. 2024 Jan 4;23(4):1426–34. doi: 10.1007/s12311-023-01644-z (PMC11269334; doi:10.1007/s12311-023-01644-z)
Supplement: Supplementary file 1 — Supplementary file1 (DOCX 16 KB) [file 12311_2023_1644_MOESM1_ESM.docx]

**Supplementary materials**

**Table 1**

Linear mixed effects model results predicting heart rate from tDCS condition, aggression and SAC (state anger change).

| *Predictors* | *β* | *SE* | *t* | *p* |
| --- | --- | --- | --- | --- |
| tDCS condition | 0.132 | 0.130 | 1.02 | 0.307 |
| aggression | 0.004 | 0.004 | 0.84 | 0.402 |
| SAC | 0.094 | 0.024 | 3.99 | **<0.001** |
| tDCS condition * aggression | -0.014 | 0.004 | -3.48 | **0.001** |
| tDCS condition * SAC | -0.028 | 0.024 | -1.20 | 0.231 |

**Table 2**

Linear mixed effects model results predicting HRV from tDCS condition, aggression and SAC (state anger change).

| *Predictors* | *β* | *SE* | *t* | *p* |
| --- | --- | --- | --- | --- |
| tDCS condition | -0.002 | 0.059 | -0.05 | 0.962 |
| aggression | 0.006 | 0.003 | 1.66 | 0.096 |
| SAC | -0.078 | 0.019 | -4.07 | **<0.001** |
| tDCS condition * aggression | 0.004 | 0.003 | 1.18 | 0.238 |
| tDCS condition * SAC | 0.030 | 0.019 | 1.59 | 0.111 |
